# Severe thrombocytopenia after trastuzumab retreatment: a case report

**DOI:** 10.1186/1756-0500-6-400

**Published:** 2013-10-04

**Authors:** Maria Simona Pino, Catia Angiolini, Luisa Fioretto

**Affiliations:** 1 Department of Oncology, Oncology Unit, Azienda Sanitaria 10 Firenze, Florence, Italy

**Keywords:** Trastuzumab, Breast cancer, Thrombocytopenia

## Abstract

**Background:**

Trastuzumab prolongs survival of human epidermal growth factor receptor 2-positive breast cancer patients in both the adjuvant and metastatic settings. Currently toxicity data are not available on retreatment of metastatic breast cancer patients who relapse after adjuvant trastuzumab. We report one patient with metastatic breast cancer who developed acute thrombocytopenia after trastuzumab infusion. This patient had trastuzumab treatment in the adjuvant setting.

**Case presentation:**

A 70-year-old Caucasian woman received a diagnosis of metastatic breast cancer four years after her initial diagnosis of locally advanced, hormone receptors-positive, human epidermal growth factor receptor 2-positive breast cancer. Trastuzumab retreatment was planned. Less than 24 hours after trastuzumab infusion, the patient was admitted to the hospital for the appearance of diffuse petechial hemorrhages and ecchymosis. The patient was confirmed to have a severe trastuzumab-induced thrombocytopenia. A rapid and complete recovery was observed after high-dose intravenous corticosteroids and immunoglobulin. No trastuzumab retreatment was attempted.

**Conclusion:**

Among the reported cases of trastuzumab-induced thrombocytopenia, this is the first report in the literature occurring in a patient retreated with trastuzumab after adjuvant therapy.

## Background

Trastuzumab is a humanized monoclonal antibody directed against the human epidermal growth factor receptor 2 (HER2). The combination of trastuzumab with chemotherapy has been shown to improve survival in both the metastatic and adjuvant settings [1-5]. Myelosuppression is rare and generally mild after trastuzumab treatment [6,7]. Nevertheless, five cases of trastuzumab-related thrombocytopenia have been reported to date [8-12]. In four cases a quick and sustained recovery of platelet count after high-dose intravenous corticosteroids and immunoglobulin was observed. Yet, a patient manifested a chronic evolution of thrombocytopenia, with refractoriness to immunosuppressive treatment. Among the reported cases of trastuzumab-induced severe thrombocytopenia, only one patient, in the adjuvant setting, did not interrupt trastuzumab. Currently, no toxicity data are available on retreatment with trastuzumab after relapse following adjuvant trastuzumab. Here, we describe a case of acute thrombocytopenia in one patient with metastatic breast cancer, which received trastuzumab several years after its adjuvant use.

## Case presentation

**Figure 1:**
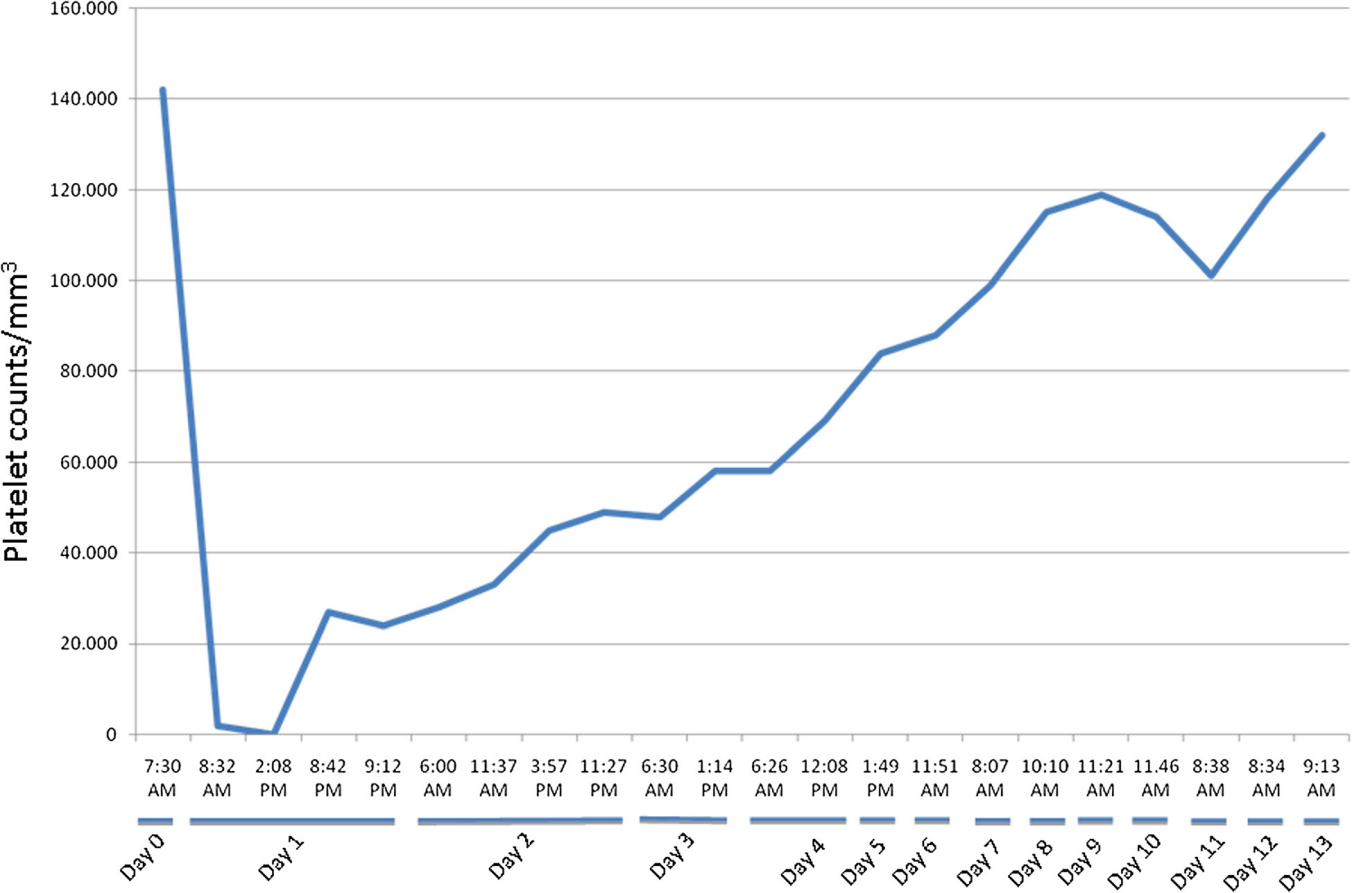
**Platelet counts before and after trastuzumab therapy.** Left y-axis represents platelet counts and X-axis represents days before and after the treatment.

**Figure 2:**
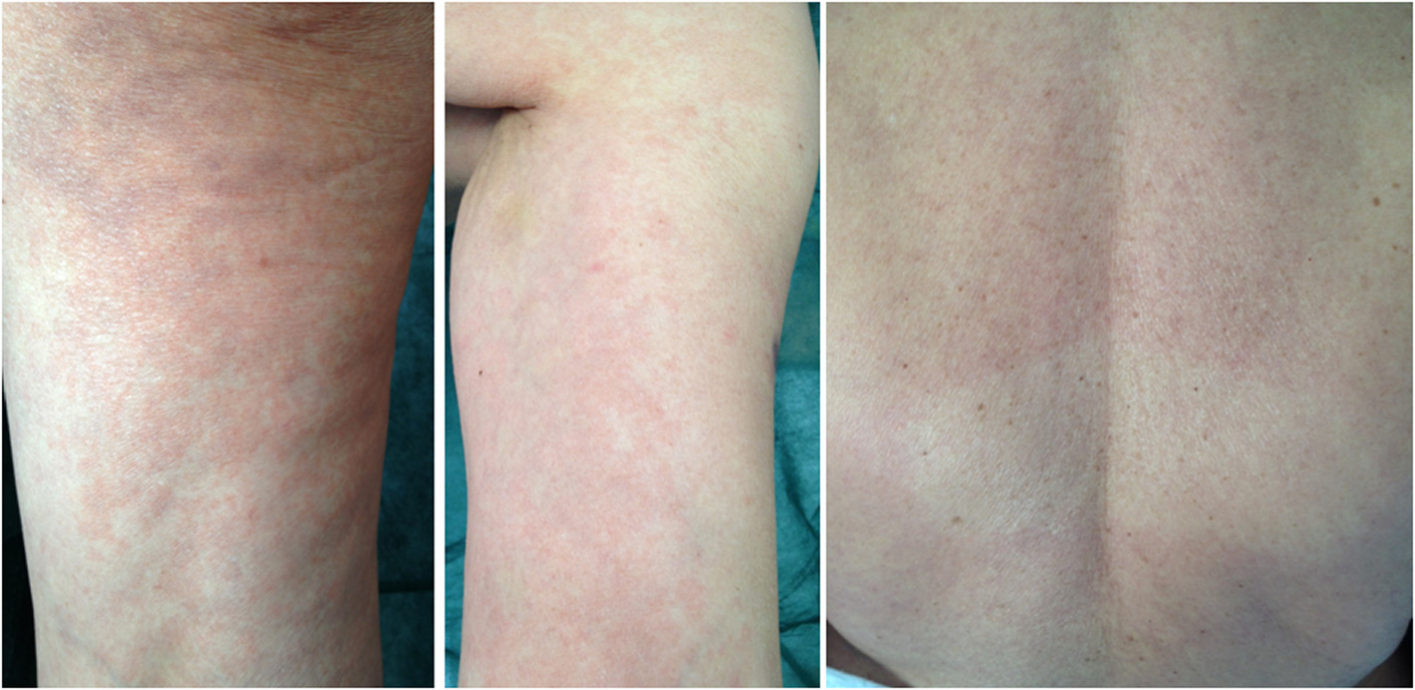
Six days after trastuzumab infusion, a pruriginous skin eczema with a generalized rash was observed, attributed to the induction of specific anti-idiotype antibodies.

A 70-year-old Caucasian woman was diagnosed with locally advanced hormone receptors-positive, HER2-positive breast cancer in 2008. She received neoadjuvant chemotherapy with a sequential anthracyclines-taxane regimen, surgery and radiotherapy. After surgery the patient received adjuvant anastrozole and trastuzumab, as per the HERceptin Adjuvant (HERA) trial [2]. Follow-up was negative until July 2012 when imaging demonstrated an extended bone relapse. A second line treatment with trastuzumab and oral vinorelbine was planned. The day of her loading dose (8 mg/kg of trastuzumab) full blood count was within normal limits Figure 1. She did not receive any heparin (either as treatment or flush), and the infusion of trastuzumab was uneventful. Less than 24 hours after the infusion, the patient turned to the emergency room for the appearance of diffuse petechial hemorrhages and ecchymosis on the lower extremities, lips and buccal mucosa. She was afebrile and cardiovascularly stable. The platelet count was 2000/mm^3^, with normal hemoglobin and leukocyte count, and negligible routine biochemistry and clotting. After 6 hours from admittance, platelet count was 0/mm^3^. High-dose immune globulin (1 g per kilogram given intravenously in 24 hours, for 5 days), methylprednisolone (1 g per kilogram given intravenously for 7 days), and platelet transfusions were administered. The patient’s prior medical history was unremarkable. Other causes of thrombocytopenia were excluded due to her normal clotting, D-dimers, immunoglobulins, renal function, bilirubin and lactate dehydrogenase as well as lack of schistocytes on blood smear and normal activity of the disintegrin and metalloproteinase with a thrombospondin type 1 motif, member 13 (ADAMTS-13). After 12 hours from admittance the platelet count started recovering. Six days after receiving trastuzumab, a pruriginous skin eczema with generalized rashes was observed, attributed to the induction of specific anti-idiotype antibodies Figure 2. The rash resolved spontaneously within 48 hours. The patient was discharged by day 13 with low dose oral prednisone. No trastuzumab retreatment was attempted.

## Conclusions

Diagnosis of drug-induced immune thrombocytopenia is usually made by exclusion. In our case, based on the clinical criteria and level of evidence elaborated by George and colleagues, the causative relationship was considered probable [13]. Indeed, treatment with trastuzumab preceded thrombocytopenia, recovery was completed and sustained after its discontinuation, and other causes of thrombocytopenia were excluded. We hypothesize that thrombocytopenia was related to preexisting drug-specific antibodies recognizing murine component of chimeric Fab fragment specific for platelet membrane glycoprotein IIIa. Clearance of the antibody-coated platelets by the mononuclear phagocytic system would be the ultimate cause of the severe thrombocytopenia. On the basis of our observation, trastuzumab retreatment calls for prudence.

## Consent

Written informed consent was obtained from the patient for publication of this Case Report and any accompanying images. A copy of the written consent is available for review by the Editor-in-Chief of this journal.

## Abbreviations

HER2: Human epidermal growth factor receptor 2; HERA: HERceptin adjuvant trial; ADAMTS: A disintegrin and metalloproteinase with thrombospondin motifs.

## Competing interests

The authors declare that they have no competing interests.

## Authors’ contributions

Conception: MSP, CA, LF; Manuscript writing: MSP; Final approval: MSP, CA, LF; Patient’s management: CA; All authors read and approved the final manuscript.

## References

[B1] Perez EA Romond EH Suman VJ Jeong JH Davidson NE Geyer CE Jr Martino S Mamounas EP Kaufman PA Wolmark N Four-year follow-up of trastuzumab plus adjuvant chemotherapy for operable human epidermal growth factor receptor 2-positive breast cancer: joint analysis of data from NCCTG N9831 and NSABP B-31 J Clin Oncol 2011 29 3366 3373 10.1200/JCO.2011.35.0868 21768458 PMC3164242

[B2] Gianni L Dafni U Gelber RD Azambuja E Muehlbauer S Goldhirsch A Untch M Smith I Baselga J Jackisch C Cameron D Mano M Pedrini JL Veronesi A Mendiola C Pluzanska A Semiglazov V Vrdoljak E Eckart MJ Shen Z Skiadopoulos G Procter M Pritchard KI Piccart-Gebhart MJ Bell R Herceptin Adjuvant (HERA) Trial Study Team Treatment with trastuzumab for 1 year after adjuvant chemotherapy in patients with HER2-positive early breast cancer: a 4-year follow-up of a randomised controlled trial Lancet Oncol 2011 12 236 244 10.1016/S1470-2045(11)70033-X 21354370

[B3] Slamon D Eiermann W Robert N Pienkowski T Martin M Press M Mackey J Glaspy J Chan A Pawlicki M Pinter T Valero V Liu MC Sauter G von Minckwitz G Visco F Bee V Buyse M Bendahmane B Tabah-Fisch I Lindsay MA Riva A Crown J Breast Cancer International Research Group Adjuvant trastuzumab in HER2-positive breast cancer N Engl J Med 2011 365 1273 1283 10.1056/NEJMoa0910383 21991949 PMC3268553

[B4] Joensuu H Bono P Kataja V Alanko T Kokko R Asola R Utriainen T Turpeenniemi-Hujanen T Jyrkkiö S Möykkynen K Helle L Ingalsuo S Pajunen M Huusko M Salminen T Auvinen P Leinonen H Leinonen M Isola J Kellokumpu-Lehtinen PL Fluorouracil, epirubicin, and cyclophosphamide with either docetaxel or vinorelbine, with or without trastuzumab, as adjuvant treatments of breast cancer: final results of the FinHer Trial J Clin Oncol 2009 27 5685 5692 10.1200/JCO.2008.21.4577 19884557

[B5] Slamon DJ Leyland-Jones B Shak S Fuchs H Paton V Bajamonde A Fleming T Eiermann W Wolter J Pegram M Baselga J Norton L Use of chemotherapy plus a monoclonal antibody against HER2 for metastatic breast cancer that overexpresses HER2 N Engl J Med 2001 344 783 792 10.1056/NEJM200103153441101 11248153

[B6] Baselga J Carbonell X Castañeda-Soto NJ Clemens M Green M Harvey V Morales S Barton C Ghahramani P Phase II study of efficacy, safety, and pharmacokinetics of trastuzumab monotherapy administered on a 3-weekly schedule J Clin Oncol 2005 23 2162 2171 10.1200/JCO.2005.01.014 15800309

[B7] Hudis CA Trastuzumab-mechanism of action and use in clinical practice N Engl J Med 2007 357 39 51 10.1056/NEJMra043186 17611206

[B8] Mantzourani M Gogas H Katsandris A Meletis J Severe thrombocytopenia related to trastuzumab infusion Med Sci Monit 2011 17 CS85 CS87 10.12659/MSM.881838 21709639 PMC3539572

[B9] Drudi F Gianni L Fantini M Ravaioli A Trastuzumab-related thrombocytopenia: always a self-limiting complication? Ann Oncol 2010 21 668 669 10.1093/annonc/mdp566 20032121

[B10] Jara Sánchez C Olier Gárate C García-Donas Jiménez J Peñalver Párraga J Drug-induced thrombocytopenia induced by trastuzumab: a special challenge in a curable disease Ann Oncol 2009 20 1607 1608 10.1093/annonc/mdp374 19633054

[B11] Parikh O Neave F Palmieri C Severe thrombocytopenia induced by a single infusion of trastuzumab Clin Breast Cancer 2008 8 285 286 10.3816/CBC.2008.n.034 18650161

[B12] Cathomas R Goldhirsch A von Moos R Drugs-induced immune thrombocytopenia N Engl J Med 2007 357 1870 1871 17985437

[B13] George JN Raskob GE Shah SR Rizvi MA Hamilton SA Osborne S Vondracek T Drug-induced thrombocytopenia: a systematic review of published case reports Ann Intern Med 1998 129 886 890 10.7326/0003-4819-129-11_Part_1-199812010-00009 9867731

